# Small Compound 6-*O*-Angeloylplenolin Induces Mitotic Arrest and Exhibits Therapeutic Potentials in Multiple Myeloma

**DOI:** 10.1371/journal.pone.0021930

**Published:** 2011-07-06

**Authors:** Ying Liu, Xiao-Qin Chen, Heng-Xing Liang, Feng-Xiang Zhang, Bo Zhang, Jie Jin, Yong-Long Chen, Yong-Xian Cheng, Guang-Biao Zhou

**Affiliations:** 1 Guangzhou Institute of Biomedicine and Health and State Key Laboratory of Biomembrane and Membrane Biotechnology, Institute of Zoology, Chinese Academy of Sciences, Beijing, China; 2 School of Life Sciences, University of Science and Technology of China, Hefei, China; 3 Department of Hematology, the Cancer Hospital, Sun Yat-Sen University, Guangzhou, China; 4 State Key Laboratory of Phytochemistry and Plant Resources in West China, Kunming Institute of Botany, Chinese Academy of Sciences, Kunming, China; 5 Department of Hematology, First Affiliated Hospital, College of Medicine, Zhejiang University, Hangzhou, China; Duke University Medical Center, United States of America

## Abstract

**Background:**

Multiple myeloma (MM) is a disease of cell cycle dysregulation while cell cycle modulation can be a target for MM therapy. In this study we investigated the effects and mechanisms of action of a sesquiterpene lactone 6-*O*-angeloylplenolin (6-OAP) on MM cells.

**Methodology/Principal Findings:**

MM cells were exposed to 6-OAP and cell cycle distribution were analyzed. The role for cyclin B1 to play in 6-OAP-caused mitotic arrest was tested by specific siRNA analyses in U266 cells. MM.1S cells co-incubated with interleukin-6 (IL-6), insulin-like growth factor-I (IGF-I), or bone marrow stromal cells (BMSCs) were treated with 6-OAP. The effects of 6-OAP plus other drugs on MM.1S cells were evaluated. The *in vivo* therapeutic efficacy and pharmacokinetic features of 6-OAP were tested in nude mice bearing U266 cells and Sprague-Dawley rats, respectively. We found that 6-OAP suppressed the proliferation of dexamethasone-sensitive and dexamethasone-resistant cell lines and primary CD138+ MM cells. 6-OAP caused mitotic arrest, accompanied by activation of spindle assembly checkpoint and blockage of ubiquitiniation and subsequent proteasomal degradation of cyclin B1. Combined use of 6-OAP and bortezomib induced potentiated cytotoxicity with inactivation of ERK1/2 and activation of JNK1/2 and Casp-8/-3. 6-OAP overcame the protective effects of IL-6 and IGF-I on MM cells through inhibition of Jak2/Stat3 and Akt, respectively. 6-OAP inhibited BMSCs-facilitated MM cell expansion and TNF-α-induced NF-κB signal. Moreover, 6-OAP exhibited potent anti-MM activity in nude mice and favorable pharmacokinetics in rats.

**Conclusions/Significance:**

These results indicate that 6-OAP is a new cell cycle inhibitor which shows therapeutic potentials for MM.

## Introduction

Multiple myeloma (MM) is a malignant proliferation of bone marrow (BM) plasma cells that produce monoclonal immunoglobulin [1]. The uncontrolled growth of myeloma cells has many consequences, including anemia, immunosuppression, osteolytic lesions, and end-organ damage. Increased BM angiogenesis is also frequently observed [2]. MM accounts for 0.8% of all cancer deaths with approximately 86,000 new cases each year worldwide [3]. The annual incidence is 1–2 per 100 000 in China and 4.3 per 100 000 people in USA [2]. The use of high-dose chemotherapy followed by autologous stem cell transplantation as well as novel agents including thalidomide, bortezomib (BOR), and lenalidomide has increased remission rates and progression-free survival [4]–[7]. However, MM remains an incurable disease in that though patients often respond to initial therapy, the disease ultimately recurs and over the course of time becomes refractory to further treatment [1]. Studies demonstrate that BM microenvironment, including BM stromal cells (BMSC) [8], paracrine signaling loops involving cytokines interleukin-6 (IL-6) and insulin-like growth factor-I (IGF-I) [9], plays pivotal roles in myeloma pathogenesis and drug resistance. Hence, novel agents targeting pathways critical to myeloma cell survival/proliferation and BM microenvironment that lead to overcome of drug resistance, remain an urgent need.

MM is a disease of cell cycle dysregulation and loss of apoptotic control. Self-renewing and non-cycling myeloma cells are both found in the BM [10]. Overexpression of cyclin D1 and D3 frequently associates with MM [11], and mutually exclusive cyclin-dependent kinase (CDK) 4/cyclin D1 and CDK6/cyclin D2 pairing inactivates retinoblastoma protein and promotes cell cycle dysregulation [12]. In addition, elevated expression of cyclin B1 (CCNB1) and the mitotic cyclin-specific ubiquitin-conjugating enzyme E2C (UBE2C) is detected in MM with chromosome abnormalities [13]. While its role in MM pathogenesis is not well understood, cyclin B1 high expression predicts a favorable outcome in patients with follicular lymphoma [14], and a cyclin B1-accumulating agent induces mitotic arrest of HCT-116 colon tumor cell line [15]. Therefore, cell cycle modulation can be a target for MM therapy [15]–[19].

**Figure 1:**
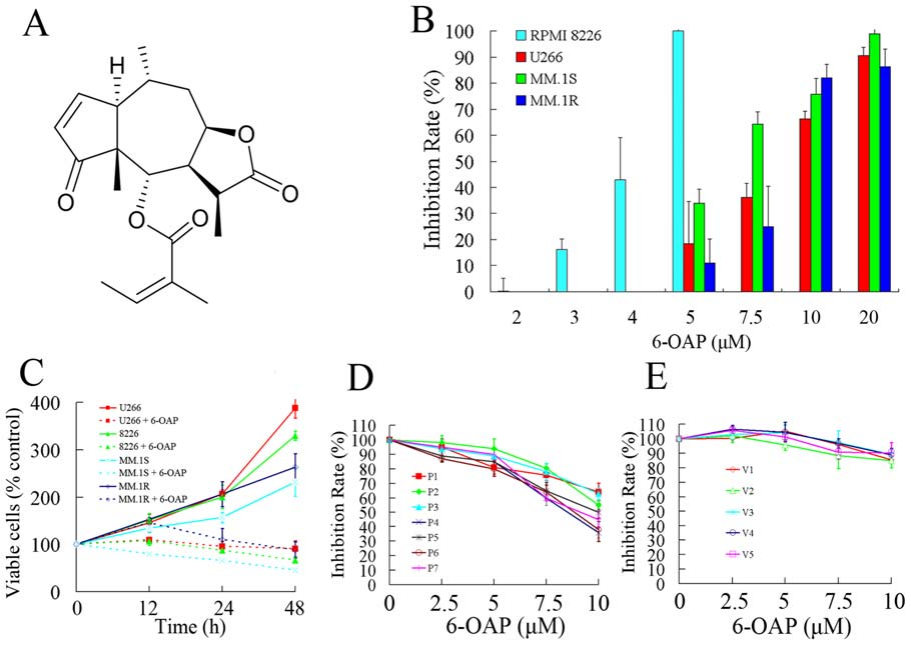
6-OAP inhibits cell proliferation of MM cells. (A) Chemical structure of 6-OAP. (B) Effects of 6-OAP on cell proliferation of RPMI 8226, U266, MM.1S, and MM.1R cells. In this experiment, cells were treated with 6-OAP at indicated concentration for 48 h, and analyzed by MTT assay. (C) MM cells were treated with or without 6-OAP, and analyzed by trypan blue exclusion assay. P values for difference between the cells treated without and with 6-OAP for 48 h: p<.0001 for U266 and RPMI 8226 cells; p = .0037 for MM.1S and p = .0043 for MM.1R cells. (D) CD138+ cells harvested from 7 MM patients (P1–P7) were treated with or without 6-OAP, and analyzed by MTT assay. (E) Peripheral blood mononuclear cells from 5 healthy volunteers (V1-V5) were co-incubated with 6-OAP at indicated concentration for 48 h, and analyzed by MTT assay.

*Centipeda minima* (L.) A.Br. is a Compositae plant distributing over East and South East Asia, Nepal and Oceania. It has been used as a medicinal herb for the treatment of headache, cough, expectoration, nasal allergy, diarrhea, malaria, and asthma in China and Korea [20], [21]. 6-*O*-angeloylplenolin (6-OAP, Figure 1A) is a sesquiterpene lactone isolated from *Centipeda minima* which is shown to have anti-bacterial and anti-protozoal activities [21]–[23]. Our preliminary data demonstrated that 6-OAP could inhibit proliferation of human colorectum, liver, stomach, lung, and skin tumor cells [24]. Recent study showed that 6-OAP could also induce apoptosis through a mitochondrial/caspase and NF-κB pathway in human HL-60 leukemia cells [25]. However, whether 6-OAP has anti-MM activity or not remains unknown. In this study, we investigated the effect of 6-OAP against human myeloma cells.

## Materials and Methods

### Ethics

Use of the samples was approved by the Institutional Review Board of Institute of Zoology, Chinese Academy of Sciences and The Cancer Hospital, Sun Yat-Sen University. All bone marrow and peripheral blood samples were obtained with written informed consent from patients at the Cancer Hospital, Sun Yat-Sen University. All animal studies were conducted according to protocols approved by the Animal Ethics Committee of the Institute of Zoology, Chinese Academy of Sciences, with the approval ID of AEC2010050804.

### Reagents

6-OAP with a purity of up to 99.5% was extracted from *Centipeda minima* (L.) as described [24]. 6-OAP was dissolved in DMSO (Sigma) at a stock solution of 10^−2^ M and stored at −20°C. Dexamethasone (Dex) was kindly provided by Dr. Hong-Qian Zhu (Department of Hematology, Nanfang Hospital Affiliated to Nanfang Medical University). Doxorubicin (Dox) was purchased from Sigma-Aldrich. BOR was attained from Millennium Pharmaceuticals Inc. IL-6, IGF-I and TNF-α were purchased from R&D systems.

### Cell culture

MM.1S, MM.1R, U266 and RPMI 8226 human MM cell lines were purchased from the American Type Culture Collection. MM.1S is a glucocorticoid (Dex)-sensitive cell line established from the peripheral blood of a patient with IgA myeloma. MM.1S cells harbor a reciprocal translocation involving 12q24.3 and 14q32.3, express leukocyte antigen DR, PCA-1, T9 and T10 antigens, and are negative for the presence of the EBV genome [26]. MM.1R is a dex-resistant variant of MM.1S. Both U266 and RPMI 8226 are plasmacytomas of B cell origin. The U266 cells produce IL-6 and are resistant to glucocorticoids, while RPMI 8226 cells produces Ig L chains but not H chain or IL-6 [27], [28]. The cells were cultured in RPMI 1640 supplemented with 10% (for U266) or 15% (for RPMI 8226, MM.1S, MM.1R) fetal bovine serum (FBS; Hyclone) and incubated in a humidified atmosphere with 5% CO_2_ at 37°C.

### Patient samples

CD138+ cells from 7 MM patients were isolated with informed consent from BM mononuclear cells by using positive immunomagnetic column separation (Miltenyi Biotech, Auburn, CA). The purity of CD138+ cells is above 97% as determined by flow cytometry. BMSCs were generated from a MM patient-derived CD138-negative BM mononuclear cells cultured for 5 weeks [29]. Peripheral blood mononuclear cells (PBMCs) from 5 healthy donors were separated by Ficoll-Hipaque density sedimentation.

### Cell proliferation and cell viability

U266, RPMI 8226 cells (1×10^4^), primary CD138+ cells, PBMCs (5×10^4^), and BMSCs (1×10^4^) were cultured in 96-well dishes and incubated without or with 6-OAP at indicated concentration for 48 h. MM.1S and MM.1R cells (2×10^4^) were seeded into 96-well dishes and pre-cultured for 24 h, then treated with 6-OAP for 48 h. Cell proliferation was determined by MTT assay. Cell viability was estimated by trypan blue dye exclusion [30].

### Analysis of cell cycle

MM cells were synchronized to G1/S boundary by a double-thymidine block. Briefly, cells were treated with 2 mM thymidine (Sigma-Aldrich) for 16 h, released into fresh medium for 9 h and subjected again to thymidine for another 16 h and then exposed to 6-OAP for indicated time points. Cells were harvested, fixed with 70% cold ethanol, incubated with RNase, and stained with propidium iodide (PI) (Sigma-Aldrich). Cell cycle distribution was analyzed by flow cytometry and CellQuest software (Becton Dickson).

### Western blotting and immunoprecipitation

Cell pellets were lysed in RIPA buffer containing 50 mM Tris pH 8.0, 150 mM NaCl, 0.1% SDS, 0.5% deoxycholate, 1% NP-40, 1 mM DTT, 1 mM NaF, 1 mM sodium vanadate, and protease inhibitors cocktail (Sigma). For immunoprecipitations, cells were lysed on ice for 15 minutes in RIPA buffer. Lysates were centrifuged. The supernatant was incubated with indicated antibodies overnight, after which protein A/G Plus beads (Santa Cruz Biotechnology) were added and incubated for 3 h. The beads were washed 4 times in RIPA buffer. Then the beads were resuspended in 1× sample buffer and boiled for 3 min. Protein extracts were quantitated and loaded on 8% to 12% sodium dodecyl sulfate polyacrylamide gel, electrophoresed, and transferred to a nitrocellulose membrane (Whatman). The membrane was incubated with primary antibody, washed, and incubated with horseradish peroxidase (HRP)–conjugated secondary antibody (Pierce). Detection was performed by using a chemiluminescent western detection kit (Cell Signaling). The antibodies used were anti-cyclin B1, anti-pCdc2 (Tyr15), anti-pStat3 (Tyr705), anti-Stat3, anti-pPDK1(Ser241), anti-PDK1, anti-pJak2 (Tyr1007/Tyr1008), anti-Jak2, anti-pHistone 3 (Ser10), anti-ubiquitin, anti-Cdc20, anti-pIκBα (Ser32/36), anti-pP65 (Ser536), anti-ERK1/2, anti-pERK1/2 (Thr202/Tyr204), anti-pJNK1/2 (Thr183/Tyr185) (Cell Signaling Technology), anti-pAkt (Ser473), anti-Akt, anti-Cdc2, anti-α-tubulin, anti-BubR1, anti-Mad2, anti-IκBα, anti-P65 (Santa Cruz Biotechnology), and anti-β-Actin (Sigma) antibodies.

### Immunofluorescence staining and confocal microscopy

Both control and 6-OAP-treated MM cells were plated on glass slides by centrifugation using a cytospin, air-dried for 5 min at room temperature, and fixed with 4% paraformaldehyde for 15 min. After a brief washing in PBS with 100 mM glycine, slides were blocked with 5% bovine serum albumin (BSA; Sigma) and 0.3% Triton X-100 in PBS for 30 min at room temperature. Microtubules were detected with indicated antibodies diluted 1∶50 in 5% BSA in PBS overnight at 4°C and FITC or rhodamine-conjugated secondary antibodies (Santa Cruz) diluted 1∶200 in 5% BSA in PBS for 1.5 h at 37°C. Cells were then washed three times with PBS. Nuclei were stained with DAPI (Sigma). Preparations were observed with a Leica TCS SP2 Spectral Confocal System, and analyzed using the LSM 3.95 software.

### Transfection of siRNA

Two siRNAs targeting cyclin B1 were designed and synthesized by Shanghai GenePharma Co., referred to as siRNA1 and siRNA2. The RNAi candidate target sequences were 5′-AAACTTTCGCCTGAGCCTATT-3′ for siRNA1 and 5′- AAGAAATGTACCCTCCAGAAA-3′ for siRNA2, respectively. Non-specific control (NC) siRNA was also purchased from GenePharma Co., with the sequences as 5'-UUCUCCGAACGUGUCACGUTT-3'.

U266 cells were transfected with siRNA using HiPerFect Transfection Reagent (Qiagen) according to the manufacturer's instructions. Cells were transfected with siRNA1, siRNA2 or NC siRNA at a concentration of 50 nM, and 6 h after transfection the cells were treated with 6-OAP at 7.5 µM for indicated time points. The cells were then harvested for cell cycle analysis, immunofluorescence staining, or lysed for Western blotting.

### Murine model

All mice used in this study were bred and maintained in a specific pathogen-free environment. BALB/c nude mice (6 to 7 weeks old) were obtained from the Shanghai Laboratory Animal Center (Shanghai, China), and maintained and monitored in a specific pathogen-free environment. All animal studies were conducted according to protocols approved by the Animal Ethics Committee of our institute. The mice were injected subcutaneously with 1×10^7^ U266 cells in 100 µL RPMI-1640 media into the right flank [31]. Treatments were started when the tumors reached a palpable size. The control group received vehicle (0.8% DMSO/18% Cremophor/8% Ethanol in normal saline), while the other two groups received intraperitoneally injection of 6-OAP (50 or 75 mg/kg per day, five times a week for 4 weeks). Caliper measurements of the longest perpendicular tumor diameters were performed twice a week to estimate the tumor volume, using the following formula: 4π/3× (width/2)^2^× (length/2), representing the 3-dimensional volume of an ellipse. Animals were sacrificed when tumors reached 2 cm or if the mice appeared moribund to prevent unnecessary morbidity to the mice. At the time of the animals' death, tumors were excised; cells were separated and subjected to immunofluorescence analysis or lyzed for Western blotting.

### Pharmacokinetic study

Eight Sprague–Dawley rats (220–250 g, female) were bought from the Laboratory Animal Center of Nanfang Medical University (Guangzhou, China), and maintained and monitored in a specific pathogen-free environment. They were fasted overnight before the experiments. All rat studies were conducted according to protocols approved by the Animal Ethics Committee of our institute. Four rats were administered with 6-OAP (30 mg/kg) by intravenous injection, and the other four rats were inoculated with 6-OAP (40 mg/kg) by intraperitoneal injection. Blood samples of 100–200 µL were collected from the orbit at the time points indicated. The plasma concentrations of 6-OAP were determined by LC-MS/MS. The pharmacokinetic parameters were obtained from the pharmacokinetic software DAS 2.0 (Drug and Statistics Version 2.0).

### Statistical analysis

All experiments were repeated at least three times and the data are presented as the mean ± SD unless noted otherwise. Differences between data groups were evaluated for significance using Student *t*-test of unpaired data or one-way analysis of variance and Bonferroni post-test. The tumor volume was analyzed with two-way ANOVA and independent sample *t* test using the software SPSS 12.0 for Windows (Chicago, IL). *P* values <.05 were considered statistically significant.

## Results

### Effects of 6-OAP on MM and normal cells

We firstly examined the growth inhibitory effect of 6-OAP on MM cell lines. By using MTT assay, we found that 6-OAP had moderate cytotoxicity to U266, RPMI 8226, MM.1S, and MM.1R cells with IC_50_ of 3.5 to 9.2 µM (Figure 1B and Table 1). By using the trypan blue exclusion assay, we found that at 5 to 7.5 µM 6-OAP markedly inhibited cell growth of U266, RPMI 8226, MM.1S, and MM.1R cells (Figure 1C). 6-OAP also suppressed cell growth of CD138+ primary cells harvested from 7 MM patients (Figure 1D), but did not drastically affect the growth of normal PBMCs from 5 healthy volunteers (Figure 1E), suggestive of a relative selectivity against MM cells.

**Table 1:** IC_50_s of 6-OAP on MM cell lines.

Cell lines	IC_50_ (µM)
U266	8.6±0.5
RPMI 8226	3.5±0.3
MM.1S	6.1±0.4
MM.1R	9.2±2.1

(The cells were treated with 6-OAP at various concentrations for 48 h, the cell proliferation was analyzed by MTT assay, and the IC_50_ was calculated using the CalcuSyn software (version 2.0, Biosoft, Cambridge, UK). Values shown are means plus or minus SD of quadruplicate determinations.)

### 6-OAP induces mitotic arrest in MM cells

**Figure 2:**
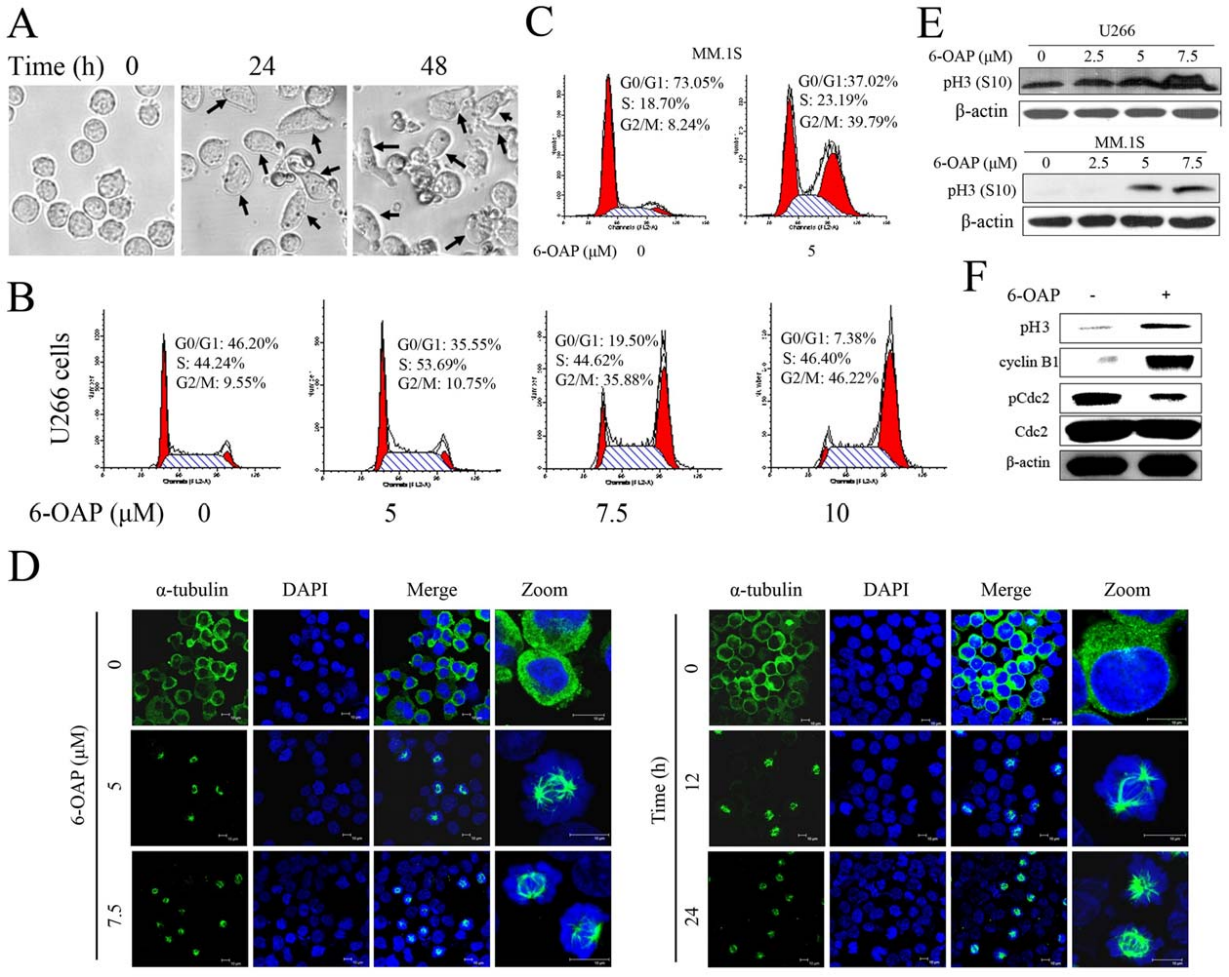
6-OAP induces mitotic arrest in MM cells. (A) U266 cells were treated with 6-OAP at 7.5 µM for 0, 24, 48 h and imaged by Leica DMI 400B microscope. (B) U266 cells were treated with 6-OAP at indicated concentrations for 48 h. Cell cycle distribution was determined by flow cytometry. (C) MM.1S cells were treated with 5 µM 6-OAP for 48 h. Cell cycle distribution was determined by flow cytometry. (D) U266 cells were treated with 6-OAP at 5 to 7.5 µM for 24 h (left panel) or at 7.5 µM for 12 to 24 h (right panel). For immunofluorescence analysis of microtubules, cells was stained with an anti-α-tubulin antibody to visualize microtubules (green) and DAPI to counter stained DNA (blue) and observed by confocal microscopy. (E) Indicated cells were treated with 6-OAP at indicated concentration for 24 h, lysed, and Western blotting was performed using antibodies indicated. (F) CD138+ primary cells isolated from 1 MM patient were treated with 6-OAP (7.5 µM) at indicated concentration for 24 h, lysed, and Western blotting was performed using antibodies indicated.

We carefully examined the morphological change of the cells, and found that after treatment with 6-OAP at 7.5 µM for 24 to 48 h, the commonly round-shaped U266 cells transformed into ellipse-shaped or spindle-shaped ones (Figure 2A). Cellular mitosis is accompanied by proper metamorphosis [32], this observation pointed to a cell cycle perturbation. We then analyzed the effects of 6-OAP on cell cycle, and the results confirmed that treatment with 6-OAP at 5 to 10 µM for 48 h led to accumulation of U266 (Figure 2B) and MM.1S (Figure 2C) cells in G2/M phase.

To distinguish the specific arrest in G2 or M phase, effects of 6-OAP on cellular microtubule networks were examined in U266 cells using a monoclonal anti-α-tubulin antibody and immunofluorescence techniques. As shown in Figure 2D, in the absence of 6-OAP the U266 cells exhibited normal microtubule staining. Interestingly, after treatment with 6-OAP at 5 to 7.5 µM for 24 h (Figure 2D, left panel) or 7.5 µM for 12 to 24 h (Figure 2D, right panel), cellular microtubules assembled to form mitotic spindle. Previous studies reported that histone 3 (H3) was phosphorylated at Ser10 during mitosis by Aurora kinase and phosphorylated H3 (pH3) could be used as a specific mitotic marker [33]. We investigated the expression of pH3, and found that treatment with 6-OAP up-regulated pH3 in U266, MM.1S (Figure 2E), and CD138+ primary cells isolated from 1 MM patient (Figure 2F). These findings indicate that 6-OAP induces mitotic arrest in MM cells.

### Accumulation of cyclin B1 is required for 6-OAP-induced mitotic arrest

**Figure 3:**
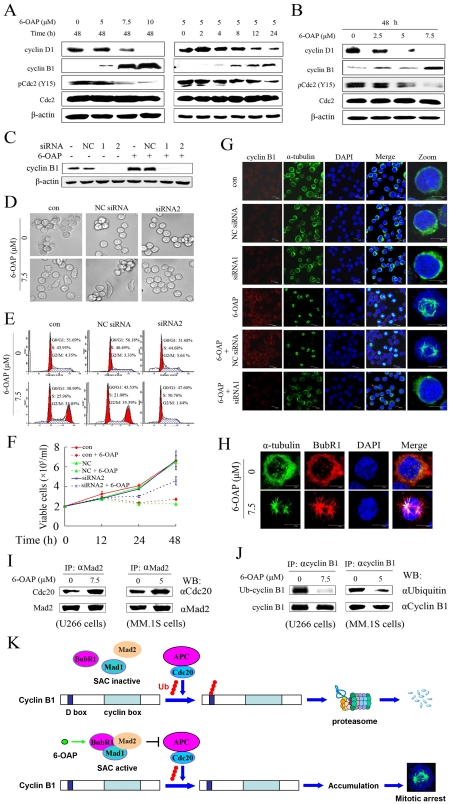
6-OAP accumulates cyclin B1 which is required for mitotic arrest. (A) U266 cells were treated with 6-OAP at indicated concentration and time points, lyzed, and Western blotting was performed using antibodies indicated. (B) MM.1S cells were treated with 6-OAP for 48 h, lyzed, and Western blotting was conducted using antibodies indicated. (C) U266 cells were transfected with 50 nM cyclin B1-specific siRNA1, siRNA2 or NC siRNA, followed by treatment without or with 6-OAP. Cells were harvested for Western blot analyses. (D through F) U266 cells were transfected with cyclin B1-specific siRNA2 or NC siRNA, followed by treatment with 6-OAP at 7.5 µM. After co-incubation with 6-OAP for 18 h, the cells were then harvested and detected by Leica DMI 400B microscope (D), or analyzed by flow cytometry for the cell cycle distribution (E). To evaluate cell growth, the cells were treated with 6-OAP for indicated time points and analyzed by trypan blue exclusion assay (F). (G) U266 cells were transfected with cyclin B1-specific siRNA1 or NC siRNA, followed by treatment with 6-OAP at 7.5 µM for 18 h. The cells were assayed by immunofluorescence analysis using an anti-cyclin B1 antibody to visualize the expression of cyclin B1 (red), an anti-α-tubulin antibody to visualize microtubules (green), and DAPI to counter stained DNA (blue). (H) U266 cells were treated without or with 7.5 µM 6-OAP for 24 h, and stained with anti-α-tubulin and anti-BubR1 antibodies to visualize microtubules (green), BubR1 (red), and DAPI to counter stained DNA (blue), and analyzed by confocal microscopy. (I and J) U266 and MM.1S cells were incubated without or with 6-OAP at indicated concentration for 24 h, lysed, and immunoprecipitation was performed followed by Western blotting using indicated antibodies. (K) Hypothetical model showing how 6-OAP causes cyclin B1 accumulation in MM cells. In eukaryocytes, APC can attach monoubiquitin to multiple lysine residues on cyclin B1, followed by polyubiquitin chain extensions linked through multiple lysine residues of ubiquitin [56]. In this simplified model only one polyubiquitin chain is shown. Ub, ubiquitin.

Mechanisms of 6-OAP-induced mitotic arrest were investigated in U266 cells which upon 6-OAP show typical metamorphosis. We tested the expression of cyclin D1, which is expressed in early G1, drives the G1/S phase transition, and is degraded in G2/M phase [34]. Our results showed that cyclin D1 was down-regulated in U266 (Figure 3A) and MM1.S cells (Figure 3B) upon 6-OAP treatment at 12 to 48 h of time course. Cyclin B1/Cdc2 complex in which Cdc2 could be dephosphorylated on Thr14 and Tyr15 [35], [36], is crucial for G2/M transition [37], [38]. We found that 6-OAP caused an increase of cyclin B1 and decrease of tyrosine-15-phosphorylated Cdc2 (pCdc2 (Y15)) in U266 (Figure 3A), MM.1S (Figure 3B) and CD138+ primary cells from MM patients (Figure 2F). To evaluate the role for cyclin B1 accumulation to play in 6-OAP-induced M phase arrest, U266 cells were transfected with siRNA1 or siRNA2 targeting cyclin B1 (Figure 3C), followed by 6-OAP treatment. Interestingly, cyclin B1 silencing abrogated 6-OAP-induced abnormal metamorphosis (Figure 3D), cell cycle arrest (Figure 3E), growth inhibition (Figure 3F) and mitotic spindle formation (Figure 3G). These data demonstrate that cyclin B1 accumulation plays a critical role in 6-OAP-triggered mitotic arrest.

In eukaryotes, the spindle-assembly checkpoint (SAC) which is comprised of checkpoint proteins including Bub1, BubR1/Mad3, Bub3, Mad1 and Mad2, is a ubiquitous safety device that ensures the fidelity of chromosome segregation in mitosis [39]. The SAC targets CDC20, a co-factor of the ubiquitin ligase anaphase-promoting complex/cyclosome (APC/C). Specifically, the SAC negatively regulates the ability of CDC20 to activate the APC/C-mediated polyubiquitylation of cyclin B and securin, thereby preventing their destruction by the 26S proteasome. Normally, ubiquitination and degradation of cyclin B inactivates Cdk1, results in exit from mitosis [39], [40]. We investigated the effects of 6-OAP on SAC component proteins. By using an anti-BubR1 antibody and immunofluorescence techniques, we found that BubR1 was assembled in 6-OAP-treated MM cells, demonstrating an activated SAC (Figure 3H). By immunoprecipitation and Western blot assays, we showed that while Mad2 bound Cdc20, 6-OAP enhanced the binding affinity in U266 and MM.1S cells (Figure 3I). Moreover, in cells upon 6-OAP, the ubiquitinated cyclin B1 (Ub-cyclin B1) was markedly decreased (Figure 3J). These results indicate that the ubiquitin-proteasome pathway-mediated cyclin B1 degradation is inhibited by the SAC activation, and 6-OAP induced-mitotic arrest is a SAC-dependent event (Figure 3K).

### 6-OAP enhances effects of other anti-MM drugs

**Figure 4:**
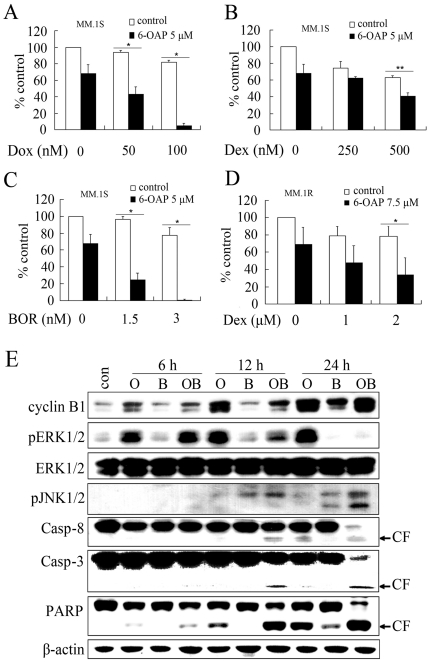
6-OAP enhances cytotoxicity of Dox, Dex and BOR to MM cells. (A through C) MM.1S cells were treated for 48 h with Dox (A), Dex (B) or BOR (C) in the presence or absence of 6-OAP at 5 µM. MTT assay was used to test the proliferation of MM.1S cells. (D) MM.1R cells were treated for 48 h with Dex in the presence or absence of 6-OAP at 7.5 µM. MTT assay was used to test the proliferation of MM.1R cells. *p<.001,** *p*<.05. (E) MM.1S cells were cultured with control media (con), 6-OAP (O, 5 µM), BOR (B, 3 nM), or 6-OAP (5 µM) plus BOR (3 nM) (OB) for indicated time points. Cells were then lysed and subjected to Western blotting using indicated antibodies. CF, cleavage fragment.

Conventional myeloma treatment will eventually fail because of intrinsic or acquired drug resistance, and only 35%–40% of MM patients respond to BOR [5], [31], [41], [42]. We investigated whether 6-OAP could enhance the effects of other anti-MM drugs in MM.1S cell line because it is sensitive to Dex, BOR and 6-OAP. The cells were treated with Dox (Figure 4A), Dex (Figure 4B), or BOR (Figure 4C) for 48 h in the absence or presence of 6-OAP (5 µM) and then analyzed by MTT assay. Our results showed that 6-OAP significantly enhanced cytotoxicity of these agents, in particular Dox and BOR (Figure 4, A through C). In MM.1R cells, Dex at 1 to 2 µM only achieved a slight inhibition rate on cell proliferation analyzed by MTT assay (Figure 4D). Interestingly, 6-OAP drastically potentiated inhibitory effect of Dex on the cells (Figure 4D).

To gain insights into the molecular mechanisms underlying the combined use of 6-OAP and BOR, several signaling pathways were investigated in MM.1S cells co-incubated with 6-OAP (5 µM)/BOR (3 nM) combinatory regimen for 6, 12, or 24 h. We found that while 6-OAP upregulated cyclin B1 expression, BOR did not enhance this effect (Figure 4E). Studies demonstrate that activation of the extracellular signal-regulated kinases 1/2 (ERK1/2) confers a drug-resistant phenotype to cancer cells [43]–[45]. In this study, we found that treatment with 6-OAP for 6 h induced an increase in phosphorylated ERK1/2 (pERK1/2) (Figure 4E). Interestingly, treatment with BOR for 12 to 24 h dramatically inhibited 6-OAP-induced pERK1/2 upregulation (Figure 4E), suggesting that BOR might be helpful for overcoming potential resistance to 6-OAP. Treatment with 6-OAP/BOR combination for 12 to 24 h upregulated phosphorylation of c-Jun N-terminal kinase (pJNK), and induced activation of caspase-8 and -3, and cleavage of casp-3 substrate PARP (Figure 4E). While cleavage of PARP was seen in an early stage (6 h), activation of casp-3 was seen in cells upon 6-OAP/BOR for 12 h (Figure 4E), suggesting that other effector caspases such as casp-7 could also be activated by the treatment protocol.

### 6-OAP overcomes the protective effects of IL-6, IGF-I, and BMSCs on MM cells

**Figure 5:**
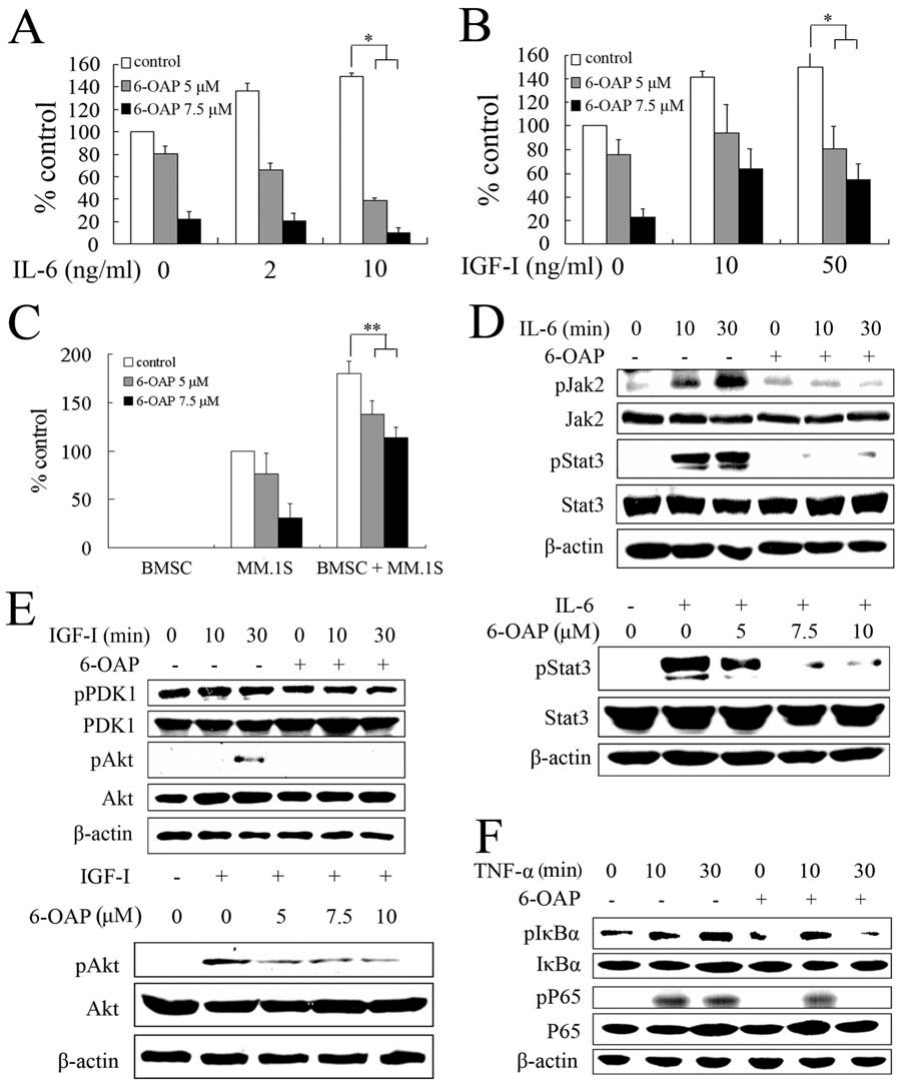
6-OAP overcomes the protective effects of IL-6, IGF-I and BMSCs on MM cells. (A and B) MM.1S cells were cultured for 48 h with indicated concentrations of 6-OAP at the presence or absence of IL-6 (A) or IGF-I (B). Cell proliferation was assessed by MTT assay. (C) MM.1S and/or BMSCs cells were cultured for 48 h with indicated concentrations of 6-OAP. Cell proliferation was assessed by MTT assay. *p<.01,** *p*<.05. (D) MM.1S cells were serum starved for 2 h, then co-cultured without or with 6-OAP at 5 µM for 12 h, followed by stimulation with IL-6 at 10 ng/ml for indicated time points (upper panel), or IL-6 at 10 ng/ml for 1 h, followed by treatment with 6-OAP at indicated concentration for 12 h (lower panel). Whole-cell extracts were prepared and examined by Western blotting using antibodies against pJak2, pStat3, Stat3 or β-actin. (E) MM.1S cells were serum starved for 2 h, then co-cultured without or with 6-OAP (5 µM) for 12 h, followed by stimulation with IGF-I at 50 ng/ml for indicated time points (upper panel), or stimulated with IGF-I at 50 ng/ml for 1 h, followed by treatment with 6-OAP at indicated concentration for 12 h (lower panel). Whole-cell extracts were prepared and examined by western blotting using antibodies against pPDK1, pAkt, Akt or β-actin. (F) MM.1S cells were serum starved for 2 h, then co-cultured without or with 6-OAP (5 µM) for 12 h, followed by stimulation with TNF-α at 5 ng/ml for indicated time points. Whole-cell extracts were prepared and examined by Western blotting using indicated antibodies.

IL-6 and IGF-I have been shown to be able to confer MM cells with resistance to therapeutics through induction of phosphatidylinositol 3-kinase (PI3-K)/Akt and/or Janus-activated kinase 2 (Jak2)/signal transducers and activators of transcription 3 (Stat3) signaling [29], [46], [47]. We therefore examined whether 6-OAP could abrogate the protective effects of IL-6 and IGF-I on MM.1S cells. We found that IL-6 at 2 to 10 ng/ml promoted proliferation of MM.1S cells detected by MTT assay, while 6-OAP at 5 to 7.5 µM drastically reversed this effect (Figure 5A). Similarly, co-incubation with exogenous IGF-I (10 to 50 ng/ml) resulted in myeloma cell expansion, and 6-OAP at 5 to 7.5 µM abolished IGF-I-caused cell proliferation (Figure 5B). We further generated BMSCs from CD138- BM mononuclear cells isolated from a de novo patient with MM [29], followed by co-incubation of the cells with MM.1S cells in the absence or presence of 6-OAP (at 5 or 7.5 µM). We found that while BMSCs facilitated MM.1S cell expansion, 6-OAP significantly inhibited this effect (Figure 5C).

We then tested the effects of 6-OAP on Jak2/Stat3 and Akt signal cascades which play a major role in the proliferation and survival of MM cells [48], [49]. Our results showed that in MM.1S cells, phosphorylated Stat3 (pStat3) and its upstream molecule, pJak2, was upregulated by treatment with IL-6 at 10 ng/ml for 10 to 30 min (Figure 5D, upper panel). Interestingly, pretreatment with 6-OAP (7.5 µM for 12 hours) markedly inhibited IL-6-induced upregulation of pJak2 and pStat3 (Figure 5D, upper panel). We further showed that the markedly upregulated pStat3 in cells upon IL-6 at 10 ng/ml was dramatically reduced by 6-OAP treatment at 5 to 10 µM for 12 h (Figure 5D, lower panel). Similarly, treatment of MM.1S cells with 6-OAP (at 7.5 µM for 12 h) inhibited IGF-I (at 50 ng/ml for 10 to 30 min, or for 1 h)-induced pAkt expression (Figure 5E, upper and lower panels). However, phosphorylation of PDK-1, a known upstream molecule of Akt, is not affected by 6-OAP (Figure 5E, upper panel). These results indicate that 6-OAP can block Jak2/Stat3 and Akt signal pathways to overcome the protective effects of IL-6 and IGF-I on MM cells, respectively, and Akt might be directly inhibited by 6-OAP.

The activation of NF-κB confers growth/survival advantage and drug resistance to MM cells in the bone marrow milieu [50], [51]. We tested whether 6-OAP inhibited this pathway stimulated by TNF-α. As can be seen in Figure 5F, we showed that in MM.1S cells 6-OAP inhibited phosphorylation of IκBα (pIκBα) and pP65 induced by TNF-α, indicating that degradation of IκBα and translocation of P65 from cytoplasm to nucleus can be blocked by 6-OAP.

### 6-OAP shows *in vivo* anti-MM activity and favorable pharmacokinetic profiles

**Figure 6:**
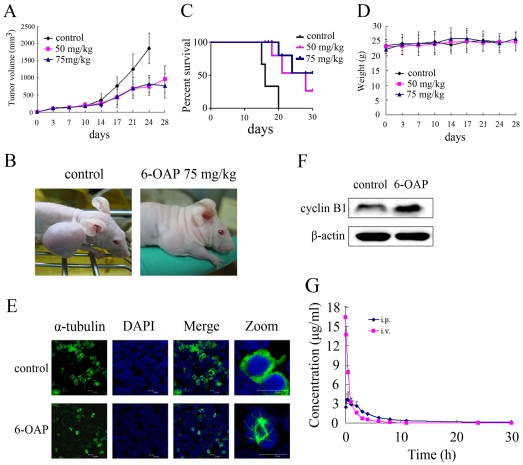
In vivo therapeutic efficacy of 6-OAP on human MM murine model. Nude mice were given subcutaneous inoculations in the right flank with 1×10^7^ U266 cells. When the tumors reached a palpable size, the mice were treated intraperitoneally with vehicle or 6-OAP (50 or 75 mg/kg, 5 times a week for 4 weeks). (A) 6-OAP significantly inhibited MM tumor growth (P<.0001, 50 or 75 mg/kg vs control). (B) Growth inhibition of subcutaneous tumors was observed in mice treated with 6-OAP. (C) Survival curve of control and 6-OAP-treated mice. (D) Treatment with 6-OAP did not affect animal body weight. (E) Tumor samples were harvested from mice treated with vehicle or 50 mg/kg 6-OAP and subjected to immunofluorescence analysis using an anti-α-tubulin antibody and DAPI. (F) Tumor tissues were harvested from mice treated with vehicle or 50 mg/kg 6-OAP, whole-tissue lysates were subjected to Western blotting using an anti-cyclin B1 antibody. (G) The concentration-time profiles of 6-OAP after intravenous (30 mg/kg) or intraperitoneal (40 mg/kg) injection of 6-OAP in Sprague-Dawley rats.

We tested the *in vivo* anti-MM efficacy of 6-OAP. To do this, nude mice were subcutaneously inoculated into the right flank with U266 or MM.1S cells, and the results showed that 90% of the mice injected with U266 cells developed a measurable tumor after a mean of 8 (6 to 13) days, while only 30% of mice inoculated with MM.1S cells developed a measurable tumor after a mean of 14 (8 to 20) days. Therefore, U266 cells were used to establish MM murine model to test the *in vivo* therapeutic efficacy of 6-OAP. Nude mice were subcutaneously inoculated into the right flank with 1×10^7^ U266 cells in 100 µL RPMI 1640 media [31]. When the tumors reached a palpable size, the mice were randomized into 3 groups (n = 10 for each group) and treated with 6-OAP at 50 or 75 mg/kg (five times a week for four weeks) or vehicle control. Animals were humanely killed when their tumors reached 2 cm in diameter or when paralysis or major compromise in their quality of life occurred. Intriguingly, we found that 6-OAP at both 50 and 75 mg/kg significantly inhibited tumor growth compared to vehicle control (*P<*.0001) (Figure 6, A and B). Consistent with these data, the survival of 6-OAP-treated mice was also prolonged compared to mice treated with vehicle control (Figure 6C). Importantly, treatment with 6-OAP did not reduce body weight of mice (Figure 6D). The tumor samples were isolated, cells were harvested, and experiments were conducted to test whether 6-OAP induced mitotic arrest and perturbed cyclin B1 expression *in vivo*. Interestingly, we showed that in samples from mice treated with 6-OAP, apparent spindle formation was detected (Figure 6E), indicating mitotic arrest of the cells. Moreover, cyclin B1 was accumulated in samples from 6-OAP-treated mice as compared to tumor tissues from control mice (Figure 6F).

We then tested the pharmacokinetic features of 6-OAP in Sprague-Dawley rats. To do this, 4 rats were administered with 6-OAP (30 mg/kg) by intravenous injection, and another 4 rats were inoculated with 6-OAP (40 mg/kg) by intraperitoneal injection. The mean plasma concentration-time profiles (n = 4) were shown in Figure 6G, and the main pharmacokinetic parameters were summarized in Table 2. We reported that in 4 rats received intravenous injection of 6-OAP at 30 mg/kg, 6-OAP in plasma achieved a peak concentration of 16.437±1.936 µg/mL (47.506±5.595 µM) at 0.033 h (1.980 min), with a t_1/2_ of 1.764 h. In 4 rats administrated intraperitoneally with 6-OAP at 40 mg/kg, 6-OAP in plasma achieved a peak concentration of 3.872±1.116 µg/mL (11.191±3.225 µM) at 0.813 h (48.780 min), with a t_1/2_ of 4.676 h. Moreover, in rats with intraperitoneal injection, the bioavailability of 6-OAP was 92.5%. These results demonstrate that administration of 6-OAP via intravenous injection as well as intraperitoneal inoculation can reach the therapeutic concentration of 6-OAP used *in vitro*, indicating the tremendous therapeutic potentials of this compound in treating human myeloma.

**Table 2:** Pharmacokinetic parameters of 6-OAP in Sprague-Dawley rats.

Administration and dosage	T_1/2_(h)	AUC_0-t_ (µg/mL·h)	T_max_(h)	C_max_ (µg/mL)	MRT_0-t_(h)	CL_z_ (L/h/kg)	BA(%)
*iv* (30 mg/kg)	1.764±0.453	14.690±1.019	0.033±0.000	16.437±1.936	1.444±0.448	2.045±0.146	—
*ip* (40 mg/kg)	4.676±0.463	18.021±2.702	0.813±0.080	3.872±1.116	5.750±1.027	2.240±0.335	92.5

AUC, Area under the plasma concentration time curve; BA, bioavailability; CL, total plasma clearance; C_max_, maximum observed plasma concentration; *iv*, intravenous injection; *ip*, intraperitoneal injection; MRT, mean residence time; T_1/2_, terminal elimination phase half-life.

## Discussion

Traditional medicines continue to provide front-line pharmacotherapy for many millions of people worldwide [52]. For malignant neoplasms such as myeloma, natural compounds can also be a source of inspiration for drug discovery. *Centipeda minima* is a medicinal herb from which three sesquiterpene lactones, 6-*O*-methylacrylylplenolin, 6-*O*-isobutyroylplenolin, and 6-OAP have been extracted. These compounds exhibit antibacterial activity against *Bacillus subtilis* and *Staphylococcus aureus*, while 6-OAP also shows anti-tumor activity on some malignant cells [24], [25]. In the present study, we report for the first time that 6-OAP exhibits moderate anti-MM activity *in vitro* and *in vivo*, and enhances the efficacy of BOR, Dox and Dex, but do not show toxicity to normal PBMCs from healthy donors and nude mice, demonstrating its therapeutic potential. Interestingly, while the widely used mitotic inhibitor paclitaxel is an extremely complex diterpene isolated from the bark of the Pacific Western yew [53], 6-OAP is a relatively simple and small compound. While the Yew tree is a limited resource, *Centipeda minima* (L.) A.Br. is rich in many countries. Furthermore, intravenous injection of 6-OAP obtains a very high plasma drug concentration, and the bioavailability of 6-OAP administrated by intraperitoneal injection reaches 92.5%. Our data thus clearly indicate the promise of 6-OAP in treating human neoplasms.

MM is a heterozygous disease, and each MM cell line has its unique characteristics. We test the anti-myeloma efficacy of 6-OAP on 4 cell lines (U266, RPMI 8226, MM.1S and MM.1R) and CD138+ primary cells harvested from MM patients. We show that upon 6-OAP, U266 cells [27], [28] exhibit typical morphological changes of mitotic arrest, so we choose this cell line to evaluate the role for cyclin B1 to play in 6-OAP-induced mitotic arrest. We employ MM.1S line in the mechanism studies for the following reasons: (1) This cell line is negative for the presence of the EBV genome [26]. (2) Results obtained from experiments using this cell line can be compared to those based on assays using MM.1R cells, if necessary. Together with MM.1R, MM.1S cell line provides a useful model to investigate the mechanisms of new agents and potential strategy to overcome Dex-resistance. (3) It is sensitive to glucocorticoid (Dex), thus represents a valuable tool to elucidate the mechanisms of action of glucocorticoids. (4) While IL-6, IGF-I and BMSCs facilitate the cells' growth and proliferation, MM.1S cells can serve as a tool to study the chemical biology of new compounds on cell-microenvironment interaction. To establish MM murine model, both U266 and MM.1S cells are inoculated into nude mice and we report that 90% of the mice injected with U266 cells develop a measurable tumor after a mean of 8 (6 to 13) days, while only 30% of mice inoculated with MM.1S cells have a palpable tumor after a mean of 14 (8 to 20) days. Therefore, U266 cells were used in our study to establish MM murine model to test the *in vivo* therapeutic efficacy of 6-OAP. The preclinical results obtained from these cellular and animal models thus provide rationales for a possible trial to test the therapeutic efficacy of 6-OAP in MM patients.

Cell cycle deregulation is a common feature for cancer, and cell cycle targeting compounds may be able to block the initiation or progression of cancer cells. For example, mitosis arresting anticancer agents, paclitaxel and vincristine, are currently used in treating solid tumors and leukemia [17]. In this work, we show that 6-OAP causes accumulation of U266 and MM.1S cells at the G2/M phase (Figure 2, B and C). We further demonstrate that 6-OAP induces mitotic arrest by observing α-tubulin staining and pH3 (S10) expression [33] in these cells upon 6-OAP treatment (Figure 2, D through F). Previous studies reported that cyclin B1 accumulates in S-phase, reaches a maximum at mitosis, and is then rapidly degraded at the metaphase/anaphase transition [54], [55]. We demonstrate that 6-OAP treatment leads to inappropriate accumulation of cyclin B1 (Figure 3, A and B). 6-OAP activates cyclin B1/Cdc2 complex by up-regulating the expression of cyclin B1 and down-regulation of pCdc2 (Y15) (Figure 3, A and B). By RNAi experiment, we show that cyclin B1 silencing leads to a decrease of 6-OAP-induced spindle-shaped cells and overcome of cell cycle arrest and growth inhibition (Figure 3, D through F). In eukaryocytes, APC attaches ubiquitin to cyclin B1 [36], [56], and the proteasomal degradation of cyclin B1 can be inhibited in mitosis by SAC [57]. Two SAC proteins BubR1 and Mad2 appear to be direct inhibitors of APC/C ubiquitin ligase [58]. Studies further show that Mad2 binds to Cdc20 which is necessary for ubiquitin ligase activity of the APC/C [59]. We therefore investigate the dynamics of SAC proteins, and discover that BubR1 is assembled in cells treated with 6-OAP, demonstrating an activated SAC (Figure 3H). By using immunoprecipitation assay, we show that 6-OAP facilitates the binding between Mad2 and Cdc20 (Figure 3I). We further report that in MM cells upon 6-OAP, the ubiquitinated cyclin B1 is markedly reduced (Figure 3J). Thus, 6-OAP induced-mitotic arrest and cyclin B1 accumulation are SAC-dependent events (Figure 3K). However, which component of SAC or other related molecule is the direct target of 6-OAP and how this small compound activates SAC activity remain obscure, and a biotin-labeled 6-OAP in combination with mass spectrometry assays will be helpful to elucidate its precise mechanisms on MM.

6-OAP abrogates the protective effects of IL-6 and IGF-I on myeloma cells through inhibition of Jak2/Stat3 and Akt signal pathways, respectively (Figure 5, A, B, D and E). Moreover, 6-OAP causes cytotoxicity even in MM cells adherent to BMSCs (Figure 5C). These results suggest that 6-OAP can overcome protection triggered by BM milieu. The activation of NF-κB confers MM cells growth, survival, and drug resistance in the BM milieu and modulates the expression of adhesion molecules on MM cells and BMSCs [50], [51], [60]. While BOR induces canonical NF-κB activation [61], we show that 6-OAP blocks TNF-α-induced upregulation of pIκBα and pP65 (Figure 5F). We report that 6-OAP-induced pERK1/2 is inhibited by BOR, and BOR-induced JNK/caspase-8 activation is markedly enhanced by 6-OAP (Figure 4E). At cellular level, 6-OAP potentiates cytotoxicity of BOR, Dex and Dox (Figure 4, A through D). In a xenograft murine model for MM, 6-OAP significantly inhibits tumor growth, while the animal body weight is not affected (Figure 6, A through C). We show that 6-OAP also induces mitotic arrest and cyclin B1 accumulation *in vivo* (Figure 6, E and F). Taken together, our data indicate that 6-OAP is a promising new cell cycle inhibitor that could be a promising anti-MM agent, and 6-OAP-based combinatory regimens including 6-OAP/BOR combination could have clinical therapeutic potentials.
